# Sustained Elevation of Resistin, NGAL and IL-8 Are Associated with Severe Sepsis/Septic Shock in the Emergency Department

**DOI:** 10.1371/journal.pone.0110678

**Published:** 2014-10-24

**Authors:** Stephen P. J. Macdonald, Shelley F. Stone, Claire L. Neil, Pauline E. van Eeden, Daniel M. Fatovich, Glenn Arendts, Simon G. A. Brown

**Affiliations:** 1 Centre for Clinical Research in Emergency Medicine, Harry Perkins Institute of Medical Research, Perth, Australia; 2 Discipline of Emergency Medicine, School of Primary, Aboriginal and Rural Health Care, University of Western Australia, Perth, Australia; 3 Emergency Department, Armadale Health Service, Perth, Australia; 4 Emergency Department, Royal Perth Hospital, Perth, Australia; University of Leicester, United Kingdom

## Abstract

**Objective:**

To identify biomarkers which distinguish severe sepsis/septic shock from uncomplicated sepsis in the Emergency Department (ED).

**Methods:**

Patients with sepsis underwent serial blood sampling, including arrival in the ED and up to three subsequent time points over the first 24 hours. Messenger RNA (mRNA) levels of 13 genes representing arms of the innate immune response, organ dysfunction or shock were measured in peripheral blood leucocytes using quantitative PCR, and compared with healthy controls. Serum protein concentrations of targets differentially expressed between uncomplicated sepsis and severe sepsis/septic shock were then measured at each time point and compared between the two patient groups.

**Results:**

Of 27 participants (median age 66 years, (IQR 35, 78)), 10 had uncomplicated sepsis and 17 had sepsis with organ failure (14 septic shock; 3 had other sepsis-related organ failures). At the time of first sample collection in the ED, gene expression of Interleukin (IL)-10 and Neutrophil Gelatinase Associated Lipocalin (NGAL) were significantly higher in severe sepsis than uncomplicated sepsis. Expression did not significantly change over time for any target gene. Serum concentrations of IL-6, IL-8, IL-10, NGAL and Resistin were significantly higher in severe sepsis than uncomplicated sepsis at the time of first sample collection in the ED, but only IL-8, NGAL and Resistin were consistently higher in severe sepsis compared to uncomplicated sepsis at all time points up to 24 h after presentation.

**Conclusions:**

These mediators, produced by both damaged tissues and circulating leukocytes, may have important roles in the development of severe sepsis. Further work will determine whether they have any value, in addition to clinical risk parameters, for the early identification of patients that will subsequently deteriorate and/or have a higher risk of death.

## Introduction

Sepsis is a significant global health problem with high rates of morbidity and mortality [1], and accounts for a significant proportion of Intensive Care Unit (ICU) admissions [2], [3]. The incidence of sepsis increases with age, and elderly patients are more likely to suffer death or permanent disability as a result of sepsis [4], [5]. Sepsis-associated organ dysfunction and shock are major contributors to poor outcome. The pathophysiology of sepsis is complex, with both pro-inflammatory and anti-inflammatory pathways activated [6]. The immune response is thought to depend upon both pathogen factors (load and virulence) and host factors (genetics, age and co-morbid disease burden).

The Emergency Department (ED) is the initial point of contact for most patients with community-acquired sepsis. An accurate assessment to identify actual or impending organ dysfunction or shock at this early stage may influence outcome since this is the major driver of mortality in sepsis. The distinction between uncomplicated sepsis (infection + SIRS) and severe sepsis/septic shock is clinically important in the ED in terms of early treatment and correct patient disposition. Measuring one inflammatory/immunological marker at a single time point has been shown to have little value [3], [7], [8], [9], [10], [11], [12], however a “panel” of biomarkers may provide better prediction of illness severity and clinical outcome [13], [14], [15], [16], [17]. A recent study validating a risk stratification tool found that measurement of five candidate biomarkers, admission lactate concentration, age and chronic disease burden was required to reliably estimate the probability of mortality in adults with sepsis. [18] Other studies of biomarker panels are limited by a number of factors including single time point sample collection and/or collection of samples at time points many hours after initial presentation. [16], [19], [20], [21]

The aim of this study was therefore to identify, in patients with uncomplicated sepsis and severe sepsis, differences in biomarkers representing key elements of the innate immune response and organ dysfunction very early in the course of disease. For candidate biomarkers, we assessed both differential gene expression in circulating peripheral blood leukocytes (PBL) and serum concentrations of expressed protein. For biomarkers differentially expressed between the two patient groups, we also aimed to explore changes over time.

## Methods

### Setting

Study participants were enrolled in our prospective, observational Critical Illness and Shock Study (CISS) between April 2011 and July 2012 in the Emergency Departments of one tertiary referral hospital and one community general hospital in Perth, Western Australia. The CISS methodology has been previously described [22]. Briefly, CISS is based on a convenience sample of ED patients recruited during rostered research nurse hours, 0700 to 2100 most days of the week. CISS enrolment criteria include physiological evidence of shock or respiratory failure.

### Ethics approval and consent

Ethics approval was obtained from the Human Research Ethics Committees at each hospital. Because the need for emergency care took priority, waiver of initial consent was approved under the provision of paragraph 2.3.6 of the National Health and Medical Research Council Ethical Conduct guidelines (2007). Once treatment was started, fully informed written consent was obtained as soon as possible and patients were given the option of declining further involvement and having all research samples collected up to that point destroyed.

### Case selection

CISS entry criteria required ‘shock’ (SBP<90, OR MAP<65 OR HR>SBP i.e. shock index>1, or lactate>4 mmol/l) or hypoxaemic respiratory failure (requirement of>6 L/min O_2_ to maintain saturations>90% or PaO_2_(mmHg)/FiO_2_ <200 if ventilated/venture mask). These physiological criteria were intentionally over-inclusive to maximize the recruitment of suitable patients in the dynamic ED environment and resulted in the inclusion of some patients with transient abnormal observations and a subsequently benign clinical course. We selected cases from the CISS database that; (i) satisfied a clinical definition of sepsis; likely or confirmed infection (identified by the clinical decision to administer intravenous antibiotics in the ED) along with two or more systemic inflammatory response syndrome (SIRS) criteria – temperature>38 or <36°C, HR>90 beats/min, RR>20 breaths/min or white cell count>12 or <4×10^9^/L, and (ii) had blood samples available from when enrolment criteria were met in ED (T0), and at least 2 subsequent time points within 24 hours of admission.

We excluded cases transferred from other hospitals, as these patients had often been unwell for an extended period of time, cases where sepsis was judged not to be the primary cause of illness and cases in which extracted mRNA failed our quality or quantity requirements (see below). A summary flowchart of the participant screening and enrolment process is shown in Figure S1.

### Case classification

To test our hypothesis of differential biomarker profiles associated with organ dysfunction, cases were reviewed by two physician investigators (SPJM, GA) and classified into two severity groups, uncomplicated sepsis and severe sepsis including septic shock. This was based on clinical and laboratory features, according to standard consensus criteria. [23] *Uncomplicated sepsis* was defined as a SOFA score of <2 and no requirement for organ support. *Severe sepsis* was defined as the presence of sepsis related organ dysfunction with a SOFA score on admission of ≥2. *Septic shock* was defined as persistence of a systolic BP <90 mmHg after a minimum 20 ml/kg isotonic crystalloid bolus, OR a serum lactate of ≥4 mmol/L. This clinical classification was undertaken separately and blinded to the laboratory analyses.

### Clinical data collection, follow up and outcome

Baseline clinical data included haemodynamic and respiratory parameters, electrolytes, renal function, full blood count, blood gas (venous or arterial) analysis and serum lactate. Charlson Comorbidity Index (CCI) [24], mortality in ED sepsis (MEDS) and sequential organ failure assessment (SOFA) scores were calculated. Participants were followed to 30 days from admission. Hospital length of stay, admission to intensive care and all cause mortality were recorded.

### Blood sampling and sample storage

Blood samples were collected as soon as practicable after enrolment criteria were met in the ED, (T0) and between 1–2, 3–6 and 12–30 hours post-T0. At each time, 2×4 ml EDTA plasma tube, 1×3.5 ml serum tube and 2×2.5 ml Blood RNA PAXgene tubes (PreAnalytiX GmbH, Switzerland) were collected. The RNA PAXgene tubes were placed immediately at 4°C then transferred to −20°C within 72 hours, before final storage at −80°C. Serum and EDTA plasma were collected and stored immediately at −80°C.

### Biomarker selection

Targets representing key arms of the innate immune response, organ dysfunction and shock were selected. These included pathogen-associated molecular pattern (PAMP) receptors that activate the innate immune system (Toll-Like Receptors TLR2 and TLR4), a general marker of immune cell activation (Urokinase Plasminogen Activator Receptor (UPAR)), pro- and anti-inflammatory cytokines and chemokines (Interleukin (IL)-6, IL-8, IL-10, Monocyte Chemotactic Protein-1 (MCP-1) and Macrophage Inhibitory Protein-1β (MIP-1β)), a marker of apoptosis (Fas ligand (FasL), the vasodilatory peptide Adrenomedullin (ADM) which may play a protective role after hypoxic tissue injury, the inflammatory biomarker Resistin that upregulates Vascular Cell Adhesion Molecule-1 (VCAM-1) expression on endothelial cells, and a marker of acute kidney injury (Neutrophil Gelatinase-Associated Lipocalin (NGAL)).

Changes in gene expression and protein levels over time were compared between patients with uncomplicated sepsis and severe sepsis/septic shock, and with a cohort of age and sex-matched healthy controls.

### Extraction and quality control of RNA

RNA was extracted using PAXgene Blood RNA Extraction Kits (PreAnalytiX GmbH, Switzerland) by automation with the Qiacube instrument (Qiagen, Australia). The purity and integrity of the RNA was assessed on a NanoDrop (Thermo Scientific, Australia) and Bioanalyzer (Agilent). Samples with RIN<7 and total RNA <1 µg were excluded. As a result, the quality of included samples was very high (median OD 260/280 ratio of 2.1 (IQR 2.0–2.1); median RIN 8.2 (IQR 7.9–8.6).

### Synthesis of cDNA

Complimentary DNA (cDNA) was synthesized using 1 µg RNA, 200 ng random primers and 10 mM dNTPs (Invitrogen Life Technologies, Australia), incubated at 65°C for 5 min. A mastermix of superscript III reverse transcriptase (200 units), first-strand buffer, 40 units of RNase inhibitor and 100 mM dithiothreitol (Invitrogen Life Technologies, Australia) were added and incubated at 25°C for 5 min then at 50°C for 50 min. Followed by heat inactivation at 70°C for 15 min. RNase H (1 unit; New England BioLabs, USA) was added and incubated for 20 min at 37°C. Storage of cDNA was at −20°C.

### Quantitative PCR (qPCR)

The measurement of mRNA levels of target genes was performed following MIQE guidelines [25]. PCR reactions were performed in a total volume of 10 µl, comprising 37.5 ng of each primer (Table S1), 0.5 µl of ResoLight Dye (Roche Diagnostics, Australia), 1 µl of 10x PCR buffer, 5 mM MgCl_2_, 0.2 mM dNTPs, 0.33 Units Platinum Taq DNA Polymerase (Invitrogen Life Technologies) and 2 µl of 1/10 cDNA. Cycling conditions were: 50°C for 2 min, 95°C for 10 min, followed by 40 cycles of 95°C for 15 sec, annealing temp (Table S1) for 15 sec and 72°C for 15 sec. A dissociation curve was established as follows: Samples were ramped from 60°C to 95°C stepwise at 0.05°C per second. Reactions were performed in triplicate and were optimized for temperature and magnesium concentration (Rotorgene 6000; Applied Biosystems, Viia 7). Dissociation profiles were used to check for single product amplification.

#### Template cloning for standard curve preparation

RNA extracted from PBLs stimulated overnight with phorbol myristate acetate (PMA) was used to prepare cDNA. Amplification of targets of interest was carried out using the same primers used for qPCR and products were ligated into pGEM-T Easy (Promega, Australia). JM109 Competent cells (Promega, Australia) were transformed with the construct, ampicillin resistant colonies were grown in liquid culture and plasmid DNA was prepared using the QIAprep Spin Miniprep Kit (Qiagen, Australia). Sequences not amplified in this manner were synthetically produced by Integrated DNA Technologies. Cloned sequences were verified on both strands by Sanger sequencing. Plasmids were linerarised with AatII (New England Biolabs, USA) and standard curves were prepared immediately prior to each run.

#### Analysis using qBase plus

Viia7 software determined Cq values using the Baseline Threshold algorithm. Three reference genes, GAPDH, HPRT and YWHAZ, were determined as appropriate to normalize Cq data using qBase plus software, v. 2.6 (Biogazelle, Belgium). Replicates that varied by greater than 0.8 Cq were excluded.

### Measuring serum protein levels (CBA/ELISA)

Serum concentrations of IL-6, IL-10, IL-8 and MCP-1 were measured by Cytometric Bead Array (CBA) Flex Sets as described previously. [26] Briefly, CBA Flex Sets employ a bead population with distinct fluorescence intensity for both APC and APC-Cy7 for each individual biomarker measured. Serum concentrations of NGAL and Resistin were measured using ELISAs from R&D Systems according to the manufacturer's instructions. Intra and interassay CVs were 6.55% and 8.77% for the Resistin ELISA and 6.74% and 8.22% for the NGAL ELISA.

### Statistical Analysis

Data are presented as median (interquartile range). Because of the skewed distribution of biomarker levels in both healthy controls and patients non-parametric tests were applied. When results were available from three groups the Kruskal-Wallis test was used, followed by application of a Bonferroni correction to post hoc multiple pairwise comparisons performed using the Wilcoxon rank-sum (Mann-Whitney) test. When results were available from two groups only, the Wilcoxon rank-sum (Mann-Whitney) test was performed. P-values presented in bold in the tables are those that remain significant after Bonferroni correction. Correlations were assessed with Spearman rank correlation with Bonferroni adjustments applied for multiple simultaneous testing. The Skillings-Mack test was used to test for differences over time within each patient group Statistical analysis was performed with Stata version 10.1 (StataCorp, College Station, Texas).

## Results

### Patients

The clinical characteristics for each group are presented in summary form in Table 1. Additional clinical data for individual participants is presented in Table S2. Of 27 patients, 10 had uncomplicated sepsis and 17 had sepsis with organ failure (14 with shock and 3 with other organ failures). As expected, patients with severe sepsis/septic shock were older, had greater comorbid burden and higher rates of ICU admission and mortality. The median time from arrival in the ED to initial blood sample collection (T0) was 58 minutes (IQR 29–95 minutes).

**Table 1:** Participant baseline clinical characteristics.

	Uncomplicated sepsis	Severe sepsis/shock	P value	All
**N**	10	17	-	**27**
**Age** (years)	43 (25, 75)	67 (59, 80)	0.04	**66 (35, 78)**
**M/F**	6/4	11/6	0.56	**17/10**
**Temp** (°C)	38.8 (38.3, 40)	37.9 (36.6, 38.8)	0.13	**38.5 (37.2, 39.9)**
**Pulse Rate/min**	111 (85, 123)	110 (87, 130)	0.65	**110 (85, 125)**
**Resp Rate/min**	22 (18, 32)	24 (18, 30)	0.98	**24 (18, 30)**
**MAP** (mmHg)	75 (69, 88)	63 (60, 77)	0.04	**69 (63, 86)**
**WCC** (x10^9^/L)	11.9 (9.4, 14.3)	12.8 (8.2, 15.3)	0.98	**12.5 (9.1, 14.7)**
**Lactate** (mmol/L)	1.5 (0.9, 2.1)	3.7 (2.5, 5.9)	<0.001	**2.5 (1.7, 4.3)**
**SOFA score**	0 (0, 1)	7 (5, 9)	<0.001	**3 (1, 8)**
**MEDS score**	3 (0, 7)	11 (8, 11)	0.002	**8 (3, 11)**
**CCI**	0 (0, 1)	1 (1, 2)	0.04	**1 (0, 2)**
**ICU admit (%)**	1 (10)	12 (71)	0.004	**13 (48)**
**Length of stay** (days)	5 (3, 6)	10 (6, 17)	0.037	**7 (4, 15)**
**Death within 30 days N (%)**	0 (0)	3 (18)	0.27	**3 (11)**

Unless otherwise stated values are median (IQR). MAP, mean arterial pressure; WCC, total White blood Cell Count; SOFA, sequential organ failure assessment; MEDS, Mortality in Emergency Department Sepsis: CCI, Charlson Comorbidity Index; ICU, Intensive Care Unit.

### Differential expression of genes in sepsis compared to healthy controls

Results are presented as a Calculated Normalised Relative Quantity (CNRQ), the relative gene expression compared to the three reference genes (GAPDH, YWHAZ, HPRT) for each sample (qBasePlus, Biogazelle). At T0, there was a significant difference in gene expression across all three study groups for all target genes except MIP-1β and UPAR (p<0.035 for all comparisons, Kruskal-Wallis) (Table 2). Compared to healthy controls, IL-10, Resistin and ADM expression was higher in both uncomplicated sepsis and those with severe sepsis/septic shock (p = 1.0×10^−4^, p = 1.9×10^−6^; p = 0.001, p = 2.4×10^−5^ and p = 0.005, p = 0.013, respectively, Mann Whitney), whereas FasL and IL-6 expression were lower (p = 0.005, p = 0.004 and p = 4.0×10^−4^, p = 5.3×10^−6^, respectively, Mann Whitney) (Table 2). Two genes were differentially expressed in severe sepsis/septic shock but not in uncomplicated sepsis; MCP-1 expression was lower than healthy controls (p = 0.002, Mann Whitney), whereas NGAL expression was higher (p = 7.0×10^−4^, Mann Whitney). Three genes were differentially expressed in uncomplicated sepsis patients but not in severe sepsis; IL-8 expression was lower than healthy controls (p = 5.0×10^−4^, Mann Whitney), whereas TLR2 and TLR4 expression was higher (p = 0.013 and p = 0004, respectively, Mann Whitney).

**Table 2:** Target gene expression at T0 in healthy controls, uncomplicated sepsis and severe sepsis/shock.

Target gene expression^#^	Healthy Controls (n = 19)	Uncomplicated Sepsis (n = 10)	Severe Sepsis/Septic Shock (n = 17)				
	Median (IQR)	Median (IQR)	Median (IQR)	p^1^	p^2^	p^3^	p^4^
IL-10	−0.75 (−0.92, −0.67)	−0.19 (−0.26, 0.005)	0.22 (−0.14, 0.48)	1.0×10^−4^	**1.0×10^−4^** *	**1.9×10^−6^**	0.035
Resistin	−0.70 (−0.93, −0.44)	−0.21 (−0.42, 0.14)	0.22 (−0.21, 0.75)	1.0×10^−4^	**0.001**	**2.4×10^−5^**	0.056
Adrenomedullin	−0.40 (−0.59, −0.006)	0.23 (−0.05, 0.44)	0.02 (−0.29, 0.30)	0.005	**0.005**	**0.013**	0.219
FasL	0.53 (0.37, 0.70)	0.08 (−0.33, 0.34)	−0.11 (−0.44, 0.44)	0.003	**0.005**	**0.004**	0.651
IL-6	0.49 (0.28, 0.54)	−0.09 (−0.33, 0.14)	−0.04 (−0.37, 0.16)	1.0×10^−4^	**4.0×10^−4^**	**5.3×10^−6^**	0.920
MCP-1	0.60 (0.43, 0.69)	0.47 (−0.33, 0.94)	−0.12 (−0.57, 0.50)	0.011	0.363	**0.002**	0.205
NGAL	−0.43 (−0.57, −0.14)	−0.56 (−0.61, −0.05)	0.38 (−0.23, 0.73)	0.001	0.819	**7.0×10^−4^**	**0.007**
IL-8	0.30 (0.21, 0.48)	−0.63 (−0.79, −0.002)	−0.26 (−0.41, 0.58)	0.003	**5.0×10^−4^**	0.064	0.108
TLR2	−0.18 (−0.35, −0.07)	0.14 (−0.13, 0.29)	−0.07 (−0.25, 0.21)	0.033	**0.013**	0.070	0.451
TLR4	−0.34 (−0.44, −0.04)	0.06 (−0.15, 0.39)	0.05 (−0.35, 0.17)	0.019	**0.004**	0.103	0.292
MIP-1β	0.25 (−0.03, 0.33)	−0.15 (−0.36, 0.26)	-0.18 (−0.54, 0.89)	0.389			
UPAR	−0.05 (−0.08, 0.20)	0.11 (−0.12, 0.43)	−0.07 (−0.42, 0.46)	0.586			

1 p value for difference across all three groups (Kruskal-Wallis)

2 p-value for the difference between uncomplicated sepsis and healthy controls (Mann Whitney)

3 p-value for the difference between severe sepsis/septic shock and healthy controls (Mann Whitney)

4 p value for the difference between uncomplicated and severe sepsis/septic shock (Mann Whitney)

* P-values in **bold** remain significant after Bonferonni correction (p<0.017)

# results are presented as Calculated Normalised Relative Quantity (CNRQ)

### Differential expression of genes in severe sepsis/septic shock compared to uncomplicated sepsis

Only NGAL had significantly higher expression at T0 in patients with severe sepsis/septic shock compared to patients with uncomplicated sepsis (p = 0.007, Mann-Whitney) (Table 2). IL-10 and Resistin mRNA levels were also elevated at T0 in severe sepsis/septic shock patients compared to those with uncomplicated sepsis but these differences were not statistically significant after Bonferroni correction (p = 0.035 and p = 0.056, respectively, Mann Whitney) (Table 2).

### Changes in gene expression over time

To determine if target gene expression changed significantly over time in patients presenting to the ED with sepsis, results were divided into four sample timing groups: T0 (time of first blood sample), T1-2 (samples collected between 51–125 minutes post-T0), T3-6 (samples collected between 165–365 minutes post-T0) and T12-30 (samples collected between 790–1605 minutes post-T0). If two samples were collected from an individual patient within these time frames, the result from the earliest time point was included for analysis. Target gene expression was stable over time in both patient groups, with the exception of IL-6 mRNA expression changing significantly over time in patients with uncomplicated sepsis (p = 0.019, Skillings-Mack) and UPAR in severe sepsis/septic shock (p = 0.024, Skillings-Mack) (Table S3).

### Serum concentrations of Resistin, NGAL, IL-6, IL-10, IL-8 and MCP-1

Based on qPCR results, we analysed serum concentrations of six potential biomarkers in both patient groups (n = 27) and age/sex matched healthy controls (n = 13). At T0, serum concentrations of Resistin, NGAL, IL-6, IL-10, IL-8 and MCP-1 were significantly different across all three study groups (p<0.02 for all comparisions, Kruskal-Wallis) (Table 3). Serum concentrations of Resistin, NGAL, IL-6 and IL-10 were higher in all sepsis cases compared to healthy controls (p≤0.003 for all comparisons, Mann Whitney) (Table 3). Patients with severe sepsis/septic shock, but not uncomplicated sepsis, had significantly higher serum concentrations of IL-8 and MCP-1 compared to healthy controls (p = 5.6×10^−6^ and p = 0.004, respectively, Mann Whitney).

**Table 3:** Serum biomarker concentrations in healthy controls, uncomplicated sepsis and severe sepsis/septic shock at T0.

Biomarker	Healthy Controls (n = 13)	Uncomplicated sepsis (n = 10)	Severe sepsis/septic shock (n = 17)				
	Median (IQR)	Median (IQR)	Median (IQR)	p^1^	p^2^	p^3^	p^4^
Resistin (ng/ml)	12.6 (8.6, 14.3)	36.5 (31.8, 51.1)	118 (88.2, 182)	1.0×10^−4^	**2.0×10^−4^**	**3.8×10^−6^**	**4.5×10^−5^**
NGAL (ng/ml)	114 (96, 135)	249 (222, 318)	476 (383, 843)	1.0×10^−4^	**0.001**	**5.6×10^−6^**	**2.5×10^−4^**
IL-6 (pg/ml)	0.0 (0.0, 7.1)	235 (99.3, 634)	5224 (592, 19,790)	1.0×10^−4^	**4.4×10^−5^**	**3.3×10^−6^**	**0.002**
IL-10 (pg/ml)	0.0 (0.0, 5.9)	19.1 (7.5, 40.2)	117 (26.3, 560)	1.0×10^−4^	**0.003**	**2.0×10^−4^**	0.018
IL-8 (pg/ml)	21.1 (18.3, 26.4)	44.3 (17.8, 205)	757 (221, 1898)	1.0×10^−4^	0.107	**5.6×10^−6^**	**9.0×10^−4^**
MCP-1 (pg/ml)	231 (163, 267)	497 (152, 1899)	2947 (259, 9722)	0.017	0.385	**0.004**	0.132

1 p value for difference across all three groups (Kruskal-Wallis)

2 p-value for the difference between uncomplicated sepsis and healthy controls (Mann Whitney)

3 p-value for the difference between severe sepsis/septic shock and healthy controls (Mann Whitney)

4 p value for the difference between uncomplicated and severe sepsis/septic shock (Mann Whitney)

* P-values in **bold** remain significant after Bonferonni correction (p<0.017)

At T0, serum concentrations of Resistin, NGAL, IL-6, IL-10 and IL-8 were significantly higher in patients with severe sepsis/septic shock compared to patients with uncomplicated sepsis (p = 4.5×10^−5^, p = 2.5×10^−4^, p = 0.002, p = 0.018 and p = 9.0×10^−4^ respectively, Mann Whitney) (Table 3). Serum concentrations of MCP-1 did not differ between the two patient groups. Serum concentrations of Resistin and NGAL at T0 correlated significantly with patient SOFA scores (R = 0.71, p = 0.011 and R = 0.67, p = 0.039, respectively, Spearman test with Bonferroni adjustment).

Serum concentrations of Resistin, NGAL and IL-8 were significantly different between the two patient groups at all time points (p<0.05 for all comparisons, Mann-Whitney) (Table 4). In patients with uncomplicated sepsis, the only statistically significant change was a decrease in IL-10 over time (p = 0.016, Skillings-Mack) (Table 4). However, in patients with severe sepsis/septic shock, there were statistically significant changes over time in serum concentrations of Resistin, IL-6, IL-10, IL-8 and MCP-1 (p = 0.024, p = 3.5×10^−6^, p = 5.0×10^−4^, p = 0.002, p = 0.049, Skillings-Mack). Serum concentrations of NGAL remained stable in patients with both uncomplicated sepsis and severe sepsis/septic shock over the first ∼24 hours after ED arrival (p = 0.119 and p = 0.237, respectively, Skillings-Mack) (Table 4).

**Table 4:** Serum biomarker levels in uncomplicated sepsis and severe sepsis/septic shock over time.

	Uncomplicated sepsis (n = 10*)	Severe sepsis/septic shock (n = 17*)			
	Median (IQR)	Median (IQR)	p^1^	p^2^	p^3^
**Resistin (ng/ml)**					
*T0*	36.5 (31.8, 51.1)	118 (88.2, 182)	**4.5×10^−5^**	0.064	0.024
*1*–*2 hours post-T0*	36.8 (27.2, 41.4)	99 (69.2, 149)	**1.0×10^−4^**		
*3*–*6 hours post-T0*	49.0 (37.6, 54.0)	135 (90.2, 161)	**7.0×10^−4^**		
*12*–*30 hours post-T0*	50.8 (29.3, 81.3)	122 (83.0, 163)	**0.011**		
**NGAL (ng/ml)**					
*T0*	249 (222, 318)	476 (383, 843)	**2.5×10^−4^**	0.119	0.237
*1*–*2 hours post-T0*	243 (187, 307)	506 (388, 791)	**2.0×10^−4^**		
*3*–*6 hours post-T0*	259 (232, 324)	534 (402, 798)	**0.003**		
*12*–*30 hours post-T0*	415 (232, 537)	607 (461, 737)	0.044		
**IL-6 (pg/ml)**					
*T0*	235 (99.3, 634)	5224 (592, 19,790)	**0.002**	0.803	3.5×10−6
*1*–*2 hours post-T0*	176 (51, 977)	4088 (430, 13,270)	**0.005**		
*3*–*6 hours post-T0*	321 (76.6, 669)	2253 (370, 6652)	0.023		
*12*–*30 hours post-T0*	82.2 (41.3, 320)	262 (106, 1635)	0.096		
**IL-10 (pg/ml)**					
*T0*	19.1 (7.5, 40.2)	117 (26.3, 560)	0.018	0.016	5×10^−4^
*1*–*2 hours post-T0*	31.6 (9.3, 78.4)	138 (28.2, 424)	0.071		
*3*–*6 hours post-T0*	12.7 (1.6, 16.2)	165 (20.5, 532)	0.031		
*12*–*30 hours post-T0*	9.8 (2.3, 18.6)	24.4 (6.5, 131)	0.095		
**IL-8 (pg/ml)**					
*T0*	44.3 (17.8, 205)	757 (221, 1898)	**9.0×10^−4^**	0.540	0.002
*1*–*2 hours post-T0*	91.7 (24.0, 167)	677 (231, 1196)	**0.002**		
*3*–*6 hours post-T0*	114 (32.2, 175)	736 (243, 974)	**0.002**		
*12*–*30 hours post-T0*	56.8 (15.1, 187)	287 (140, 451)	**0.011**		
**MCP-1 (pg/ml)**					
*T0*	497 (152, 1899)	2947 (259, 9722)	0.132	0.591	0.049
*1*–*2 hours post-T0*	588 (107, 1471)	2115 (513, 6367)	0.071		
*3*–*6 hours post-T0*	579 (156, 783)	1654 (647, 2722)	0.036		
*12*–*30 hours post-T0*	426 (90.0, 1239)	517 (268, 1646)	0.335		

1 p value for the difference between uncomplicated and severe sepsis/septic shock (Mann Whitney). P values in **bold** remain significant after Bonferroni correction (p<0.013).

2 p value for change over time for uncomplicated sepsis (Skillings Mack)

3 p value for change over time for severe sepsis/septic shock (Skillings Mack)

*At T0 and 1–2 hours post-T0, data was available from n = 10 uncomplicated sepsis and n = 17 severe sepsis/septic shock patients, at 3–6 hours post-T0, data was available from n = 8 uncomplicated sepsis and n = 17 severe sepsis/septic shock and at 12–30 hours post-T0 data was available from n = 6 uncomplicated sepsis and n = 13 severe sepsis/septic shock.

### Correlation of mRNA expression and serum protein concentrations

When comparing all samples from all patients at all timepoints, only Resistin and NGAL mRNA levels correlated with serum protein concentrations (R = 0.52, p<0.001 and R = 0.36, p = 0.022, respectively, Spearmans with Bonferroni adjustment). Serum levels of all biomarkers measured correlated positively with each other with p<0.003 for all comparisons except MCP-1 versus IL-10 where the p-value was 0.03.

## Discussion

In this study we used quantitative PCR to identify potential candidate genes differentially expressed in circulating leucocytes early in the ED in severe sepsis compared to uncomplicated sepsis and healthy controls. We then measured serum protein levels at multiple time points over the first 24 h of hospital stay, and identified consistently significant differences in levels of Resistin, NGAL and IL-8 between the groups at all time points.

Sepsis is a dynamic condition and patients present to the ED at various stages in their illness evolution. An essential task is to risk stratify patients to direct timely care and correct disposition. Biomarkers of organ dysfunction may more accurately detect patients with severe sepsis or shock before this manifests either clinically or with conventional markers such as creatinine or lactate. Our study obtained serial blood samples commencing early in the ED and over the subsequent 24 hours. We found that while expression of our chosen target genes in PBL did not significantly change over time, almost all of the measured proteins in serum did, specifically in the severe sepsis/septic shock group. This has clinical and research implications since timing of sampling may be critical. For example, if patients are recruited into studies in the ICU, measured levels of cytokines may be markedly different to those on arrival prior to resuscitation. A reliable serum marker for severe sepsis should differentiate patients with organ dysfunction/shock regardless of the timing of sampling and resuscitation status of the patient. Of the six serum proteins we measured based upon the results of our qPCR analysis, only three – Resistin, NGAL and IL-8 differentiated severe sepsis from uncomplicated sepsis at all time points and only NGAL remained stable over time in both patient groups. It is unlikely that any single marker will be sufficiently accurate for clinical purposes. For this reason it is suggested that a multi-marker “panel” may be more useful. [14]

Resistin, first described in 2001 as an adipocyte-secreted hormone causing insulin resistance in type-2 diabetes, has since been found to be an important pro-inflammatory cytokine in humans, secreted principally by monocytes and epithelial cells. [27], [28] In a study of patients with severe sepsis and septic shock, resistin was found to correlate strongly with disease severity, as well as with levels of inflammatory cytokines, lactate D-dimer and creatinine. [29] Another study found that resistin levels were higher among ICU patients with sepsis compared to non-infected controls, and this was unrelated to preexisting diabetes or obesity. [30]

NGAL, a member of the lipocalin family of proteins, is expressed by neutrophils and a number of epithelial cells. It has emerged as an early marker of acute kidney injury in a range of critical illness settings, including in sepsis. [31], [32] Among ED patients with sepsis elevated NGAL on admission was predictive of subsequent renal injury. [33] In a multi-centre study a biomarker panel of NGAL along with IL-1ra and protein C was predictive of severe sepsis, septic shock and death among ED patients with suspected sepsis. [14] Analysis of gene expression patterns from blood samples from septic and non-septic patients in a meta-analysis has also suggested that NGAL is a strong candidate gene for predicting SIRS patients that may progress to sepsis. [34]

A defining characteristic of severe sepsis may be recruitment of circulating immune cells to produce inflammatory mediators, in addition to local tissue production. It is notable that, for both NGAL and resistin, as well as serum concentrations being higher, gene expression was also greater in circulating leukocytes of patients with severe sepsis compared to uncomplicated sepsis.

The serum levels of individual cytokines are expected to vary enormously between individuals due to differential timing of release, metabolism and the complex interplay between the vast array of biomarkers released during sepsis. [35] In a recent study, Lvovschi et al determined that cytokine levels either individually or in combination do not appear useful for differentiating severe sepsis from uncomplicated sepsis in the ED. [36] We found that, although serum levels of IL-6, IL-10 and IL-8 were significantly elevated in severe sepsis/septic shock compared to uncomplicated sepsis up to 6–8 hours post-ED arrival, significant changes in serum levels over time, especially in patients with severe sepsis, means clinical use of these markers as a routine test to predict sepsis severity or progression may be limited.

Notable strengths of this study are the recruitment of patients very early in the course of their hospital management, with serial sampling over time. As well as addressing the issue of ‘lead time’ when patients are recruited into biomarker studies in ICU, often many hours after presentation, we also included patients not admitted to the ICU, reflecting real-world experience of sepsis in the ED. We approached the selection of biomarkers by two methods, using qPCR to identify differential gene activation in leucocytes between two clinically relevant phenotypic groups, then confirming these results by measuring the serum protein products of those genes. There are also several limitations to the study. Patients were selected by convenience sampling and the availability of sufficient quality mRNA for qPCR analysis. In addition, the study sample was a subgroup of a larger ED critical illness study and may not necessarily be representative of the ED sepsis population. Our selection of serum markers was informed by the results of the qPCR analysis of leucocyte mRNA, meaning we may have excluded important markers produced predominantly by tissues. The small number of patients and multiple statistical tests increases the risk of chance ‘positive’ findings of association between groups. The small numbers also precluded undertaking any regression analyses; given the high degree of correlation between the markers this is necessary to determine the independent predictive value of any given marker. Finally, when interpreting the analyses over time it is important to note that not all patients had complete samples at all time points which may have led to bias in the later time-point comparisons.

Our findings indicate a potential role for IL-8, Resistin and NGAL to differentiate patients with severe sepsis from those with a more benign course, however whether this might be of any use in addition to clinical parameters is unknown. The next step is to attempt to replicate these findings in a larger prospective cohort and so refine and validate a panel of biomarkers which will complement clinical assessment to identify high risk patients in the ED.

## Supporting Information

Figure S1 **A summary flowchart of the participant screening and enrolment process.**
(DOCX)

Table S1 **Primer sequences for qPCR expression analysis.**
(DOCX)

Table S2 **Clinical characteristics.**
(DOCX)

Table S3 **Target gene expression in uncomplicated and severe sepsis over time by qPCR.**
(DOCX)
