# Supplementary material for: Sustained Elevation of Resistin, NGAL and IL-8 Are Associated with Severe Sepsis/Septic Shock in the Emergency Department
Source: PLoS One. 2014 Oct 24;9(10):e110678. doi: 10.1371/journal.pone.0110678 (PMC4208806; doi:10.1371/journal.pone.0110678)
Supplement: Figure S1 — A summary flowchart of the participant screening and enrolment process. (DOCX) [file pone.0110678.s001.docx]

6318 screened for CISS enrolment

70 excluded

40 less than 3 sample time points

30 insufficient quality or quantity mRNA

27 enrolled in study

5972 Not enrolled

5948 Did not meet enrolment criteria

24 Declined consent

346 enrolled in CISS

249 excluded

244 not sepsis

5 transfer from other hospital

97 with sepsis
