# Supplementary material for: Sustained Elevation of Resistin, NGAL and IL-8 Are Associated with Severe Sepsis/Septic Shock in the Emergency Department
Source: PLoS One. 2014 Oct 24;9(10):e110678. doi: 10.1371/journal.pone.0110678 (PMC4208806; doi:10.1371/journal.pone.0110678)
Supplement: Table S1 — Primer sequences for qPCR expression analysis. (DOCX) [file pone.0110678.s002.docx]

Table S1. Primer sequences for qPCR expression analysis.

| **Target** | **Forward Primer 5’-3’** | **Reverse Primer 5’-3’** | **Amplicon Length bp** | **Tm^0^C** |
| --- | --- | --- | --- | --- |
| ADM | CCGGGCTCGCTGACGTGAAG | CCGGACTGCTGTCTTCGGGG | 96 | 65 |
| MCP-1 | GCTCGCTCAGCCAGATGCAATCA | CGCGAGCCTCTGCACTGAGA | 85 | 65 |
| MIP-1B | CGCGAGCCTCTGCACTGAGA | GGCTGGGAGCAGAGGCTGCT | 124 | 67 |
| FASLG | ATAGGCCACCCCAGTCCACCC | TGGACTTGCCTGTTAAATGGGCCAC | 70 | 67 |
| IL10 | GGCTACGGCGCTGTCATCGATT | GCATTCTTCACCTGCTCCACGG | 70 | 64 |
| IL6 | GCAGCAAAGAGGCACTGGCAGAA | CCAGGCAAGTCTCCTCATTGAATCC | 99 | 68 |
| IL8 | TTCCAAGCTGGCCGTGGCTCT | TTGGCAAAACTGCACCTTCACACA | 74 | 67 |
| NGAL | ACGGGAGAACCAAGGAGCTGACT | ACACTGGTCGATTGGGACAGGGA | 113 | 66 |
| RETN | CTTGCCCCCGAGGCTTCGC | CGCTCCGGTCCAGTCCATGC | 119 | 66 |
| TLR2 | AGCAGCAAGCACTGGCCAAAGT | GCTGGCCACGCACATGGGAT | 81 | 67 |
| TLR4 | GCCCTGCGTGGAGGTGGTTCC | GAGAAGGGGAGGTTGTCGGGGAT | 84 | 66 |
| UPAR | CACCGGCACTCACGAACCGAAA | CCCAGGTGGGCATGTTGGCAC | 84 | 67 |
| GAPDH | TCTCTGCTCCTCCTGTCGA | CCCAATACGACCAAATCCGTTG | 119 | 60 |
| HPRT1 | TGACACTGGCAAAACAATGCA | GGTCCTTTTCACCAGCAAGCT | 94 | 60 |
| YWHAZ | ACTTTTGGTACATTGTGGCTTCAA | CCGCCAGGACAAACCAGTAT | 94 | 60 |

Full gene sequences were obtained from NCBI Reference Sequence database. Primers were designed across exon boundaries where possible, Oligo Calc was used to ensure the absence of secondary structures such as hairpins, 3’complimentarity and self complimentarity. MFE primer 2 and BLAST were used to determine primer specificity. Ensembl genome browser was used to examine transcripts for the presence/absence of multiple transcripts and optimal primer locations. Primer purification was by standard desalting. Samples were assessed for inhibition and contamination by amplification of serial dilutions of RNA, a no RT control and a no template were included in every run.
