# Supplementary material for: Sustained Elevation of Resistin, NGAL and IL-8 Are Associated with Severe Sepsis/Septic Shock in the Emergency Department
Source: PLoS One. 2014 Oct 24;9(10):e110678. doi: 10.1371/journal.pone.0110678 (PMC4208806; doi:10.1371/journal.pone.0110678)
Supplement: Table S2 — Clinical characteristics. (DOCX) [file pone.0110678.s003.docx]

Table S2. Clinical characteristics

| **Group** | **Age/Sex** | **Diagnosis** | **Organism*** | **Source** | **LOS (days)** | **Outcome 28 days** | **Clinical Summary** |
| --- | --- | --- | --- | --- | --- | --- | --- |
|  |  |  |  |  |  |  |  |
| 1 | 78 M | **Hepatic abscess** |  |  | 21 | Alive | Initial admission with presumed pneumonia, transferred to ICU for observation due to persistent tachycardia on day 3. CT scan – hepatic abscess - drained percutaneously. Discharged on IV antibiotics. |
| 1 | 83 M | **Wound infection** | *S Pneumonia* | Blood | 5 | Alive | Infected tracheostomy site. IV antibiotics on ward. |
| 1 | 73 M | **Viral pneumonitis** |  |  | 4 | Alive | Fever cough and dyspnea. Treated with antibiotics and oseltamivir on ward. |
| 1 | 25 F | **Mastitis** |  |  | 4 | Alive | Fever and headache. Initial workup for meningitis negative. |
| 1 | 19 M | **Pneumonia** |  |  | 1 | Alive | Lobar pneumonia – initial IV antibiotics then discharged home. |
| 1 | 35 M | **Skin abscess** |  |  | 3 | Alive | Perianal abscess treated with IV antibiotics on ward. |
| 1 | 54 M | **Skin abscess** | *S Aureus* | Pus | 2 | Alive | Infected skin wound – IV antibiotics on ward. |
| 1 | 22 F | **Skin abscess** | *S Aureus* | Blood | 15 | Alive | Intravenous drug user – *S Aureus* bacteraemia without endocarditis. IV antibiotics on ward. |
| 1 | 27 F | **Pyelonephritis** | *Enterococcus* | Urine | 5 | Alive | Acute pyelonephritis – IV antibiotics on ward. |
| 1 | 52 F | **Pneumonia** |  |  | 6 | Alive | Hypostatic pneumonia due to multiple sclerosis. IV antibiotics on ward. |
|  |  |  |  |  |  |  |  |
| 2 | 85 M | **Cellulitis** |  |  | 12 | Died | Admitted with bilateral cellulitis with respiratory/ renal failure. Day 10 developed acute small bowel perforation and elected for non-operative management. Palliated and died. |
| 2 | 60 M | **Pneumonia** |  |  | 2 | Alive | Atypical pneumonia with acute kidney injury (creatinine 225umol/L) – responded to fluid resuscitation on ward. |
| 2 | 56 M | **Necrotizing fasciitis** |  |  | 32 | Alive | Rapidly progressive forearm cellulitis requiring surgical debridement. Intubated for respiratory distress day 2 of admission. Influenza isolated from sputum by PCR. |
| 2 | 73 F | **Urosepsis** | *E Coli* | Urine | 6 | Alive | Septic shock on background of hepatic carcinoma. Fluids/antibiotics and vasopressors on ward. |
| 2 | 67 M | **Cellulitis** |  |  | 19 | Alive | Septic shock requiring noradrenalin infusion in ICU. Background of lymphoma (in remission). |
| 2 | 66 F | **Urosepsis** |  |  | 19 | Alive | Altered conscious state with fever and high lactate. |
| 2 | 80 M | **Cholecystitis** | *E Coli* | Blood | 14 | Alive | Gram negative sepsis with shock requiring vasopressors in ICU. |
| 2 | 35 F | **Pneumonia** | *S Pneumonia* | Sputum | 10 | Alive | Lobar pneumonia with septic shock requiring vasopressors in ICU |
| 2 | 59 M | **Pneumonia** | *S Pneumonia* | Sputum | 6 | Alive | Lobar pneumonia with septic shock requiring vasopressors in ICU. |
| 2 | 86 M | **Urosepsis** | *K Pneumonia* | Urine & blood | 7 | Alive | Nursing home resident with gram-negative sepsis and high lactate. Managed on ward. |
| 2 | 74 F | **Septic shock presumed from radiation colitis** | *S Pyogenes* | Blood | 7 | Alive | Septic shock requiring vasopressors in ICU. Radiotherapy for cervical carcinoma. |
| 2 | 85 M | **Gram negative sepsis** | *P Aeruginosa* | Blood | 1 | Died | Nursing home resident with gram-negative sepsis. Managed in ward. Died day 2. |
| 2 | 66 F | **Urosepsis** | *E Coli* | Urine and blood | 8 | Alive | Acute renal failure with septic shock requiring vasopressors in ICU. |
| 2 | 80 M | **Gram negative sepsis** | *Capnocytophaga* | Blood | 18 | Alive | Gram-negative sepsis with multi-organ failure. |
| 2 | 33 M | **Septic shock** | *S Pyogenes* | Blood | 13 | Alive | Intravenous drug user, multiple skin lesions, multi-organ failure managed in ICU. |
| 2 | 71 F | **Meningococcal sepsis** | *N Meningitis* | Blood | 17 | Died | Shock and progressive multi-organ failure. Died day 17 in ICU. |
| 2 | 52 M | **Gastroenteritis** |  |  | 6 | Alive | Diarrheal illness and septic shock requiring vasopressors in ICU. Cultures negative. |

Group 1 – Infection with SIRS/uncomplicated sepsis. Group 2 – severe sepsis/septic shock. *Organism isolated from sample collected within first 24h of admission.
