# Supplementary material for: Sustained Elevation of Resistin, NGAL and IL-8 Are Associated with Severe Sepsis/Septic Shock in the Emergency Department
Source: PLoS One. 2014 Oct 24;9(10):e110678. doi: 10.1371/journal.pone.0110678 (PMC4208806; doi:10.1371/journal.pone.0110678)
Supplement: Table S3 — Target gene expression in uncomplicated and severe sepsis over time by qPCR. (DOCX) [file pone.0110678.s004.docx]

Table S3. Target gene expression in uncomplicated and severe sepsis over time by qPCR

| Target gene expression | Uncomplicated sepsis (n=10)* | Severe sepsis/septic shock (n=17)* |  |  | |
| --- | --- | --- | --- | --- | --- |
|  | Median (IQR) | Median (IQR) | p^1^ | p^2^ | p^3^ |
| **Adrenomedullin**  *T0*  *1-2 hours post-T0*  *3-6 hours post-T0*  *12-30 hours post-T0* | 0.23(-0.05-0.44)^#^  0.31(-0.16-0.44)  0.13(-0.49-0.35)  0.20(-0.02-0.78) | 0.02 (-0.29-0.30)  -0.04(-0.22-0.12)  -0.01(-0.31-0.33)  0.17(-0.18-0.21) | 0.219  0.077  0.949  0.465 | 0.532 | 0.338 |
| **MCP-1**  *T0*  *1-2 hours post-T0*  *3-6 hours post-T0*  *12-30 hours post-T0* | 0.47(-0.33-0.94)  0.19(-0.57-0.72)  0.19(-0.34-0.90)  0.20(-0.52-0.76) | -0.12(-0.57-0.50)  -0.27(-0.96-0.11)  -0.33(-0.45-0.16)  -0.45(-0.67-0.62) | 0.205  0.133  0.197  0.394 | 0.210 | 0.097 |
| **MIP-1β**  *T0*  *1-2 hours post-T0*  *3-6 hours post-T0*  *12-30 hours post-T0* | -0.15(-0.36-0.26)  -0.33(-0.41-0.77)  -0.23(-0.41- -0.02)  -0.03(-0.23-0.56) | -0.18(-0.54-0.89)  -0.42(-0.64-0.29)  0.15(-0.42-0.25)  0.33(-0.13-0.57) | 0.880  0.243  0.439  0.685 | 0.742 | 0.138 |
| **FasL**  *T0*  *1-2 hours post-T0*  *3-6 hours post-T0*  *12-30 hours post-T0* | 0.08(-0.33-0.34)  -0.03(-0.24-0.35)  -0.06(-0.46-0.17)  0.62(0.05-0.76) | -0.11(-0.44-0.44)  -0.50(-0.88-0.13)  0.08(-0.15-0.19)  -0.14(-0.4-0.11) | 0.651  0.133  0.302  0.028 | 0.597 | 0.305 |
| **IL-6**  *T0*  *1-2 hours post-T0*  *3-6 hours post-T0*  *12-30 hours post-T0* | -0.09(-0.33-0.14)  0.11(-0.44-0.29)  -0.13(-0.35-0.11)  -0.09(-0.17-0.37) | -0.04(-0.37-0.16)  -0.27(-0.54-0.09)  -0.20(-0.30-0.15)  -0.01(-0.04-0.21) | 0.920  0.243  0.747  0.372 | 0.019 | 0.099 |
| **IL-10**  *T0*  *1-2 hours post-T0*  *3-6 hours post-T0*  *12-30 hours post-T0* | -0.19(-0.26-0.005)  0.04(-0.19-0.27)  0.05(-0.37-0.24)  0.30(-0.28-0.43) | 0.22(-0.14-0.48)  0.18(0.10-0.42)  0.37(0.15-0.52)  -0.07(-0.15-0.57) | 0.035  0.102  0.033  0.570 | 0.168 | 0.767 |
| **IL-8**  *T0*  *1-2 hours post-T0*  *3-6 hours post-T0*  *12-30 hours post-T0* | -0.63(-0.79- -0.002)  -0.41(-0.62-0.09)  -0.25(-0.87- -0.03)  -0.30(-0.35-0.64) | -0.26(-0.41-0.58)  -0.06(-0.51-0.20)  -0.07(-0.36-0.72)  0.07(-0.24-0.73) | 0.108  0.367  0.175  0.465 | 0.317 | 0.427 |
| **NGAL**  *T0*  *1-2 hours post-T0*  *3-6 hours post-T0*  *12-30 hours post-T0* | -0.56(-0.61- -0.05)  -0.15(-0.61-0.26)  -0.27(-0.60-0.21)  -0.21(0.67-0.51) | 0.38(-0.23-0.73)  0.30(-0.28-0.76)  0.38(-0.63-0.67)  -0.15(-0.44-0.75) | **0.007**  0.102  0.366  0.570 | 0.096 | 0.462 |
| **Resistin**  *T0*  *1-2 hours post-T0*  *3-6 hours post-T0*  *12-30 hours post-T0* | -0.21(-0.42-0.14)  0.02(-0.56-0.55)  0.14(-0.56-0.35)  0.22(-0.28-0.40) | 0.22(-0.21-0.75)  0.42(-0.29-0.74)  0.27(-0.20-0.78)  0.41(-0.73-0.66) | 0.056  0.193  0.273  0.570 | 0.873 | 0.889 |
| **TLR2**  *T0*  *1-2 hours post-T0*  *3-6 hours post-T0*  *12-30 hours post-T0* | 0.14(-0.13-0.29)  0.14(-0.17-0.27)  0.21(-0.04-0.45)  0.02(-0.24-0.39) | -0.07(-0.25-0.21)  -0.04(-0.35-0.02)  -0.17(-0.35-0.27)  0.04(-0.05-0.50) | 0.451  0.030  0.175  0.465 | 0.877 | 0.542 |
| **TLR4**  *T0*  *1-2 hours post-T0*  *3-6 hours post-T0*  *12-30 hours post-T0* | 0.06(-0.15-0.39)  0.15(0.01-0.33)  0.21(-0.30-0.36)  -0.17(-0.33-0.18) | 0.05(-0.35-0.17)  -0.05(-0.30-0.21)  0.06(-0.44-0.22)  -0.17(-0.34-0.60) | 0.292  0.077  0.156  0.935 | 0.329 | 0.848 |
| **UPAR**  *T0*  *1-2 hours post-T0*  *3-6 hours post-T0*  *12-30 hours post-T0* | 0.11(-0.12-0.43)  0.25(-0.40-0.40)  0.17(-0.18-0.46)  0.17(-0.02-0.32) | -0.07(-0.42-0.46)  -0.26(-0.57-0.13)  0.07(-0.55-0.29)  0.27(-0.18-0.33) | 0.340  0.133  0.302  0.935 | 0.606 | 0.024 |

^1^p value for the difference between uncomplicated and severe sepsis/septic shock (Mann Whitney). P values in **bold** remain significant after Bonferroni correction (p<0.013)

^2^p value for change over time for uncomplicated sepsis (Skillings Mack)

^3^p value for change over time for severe sepsis/septic shock (Skillings Mack)

*At T0, data was available from n=10 uncomplicated sepsis and n=17 severe sepsis/septic shock, at 1-2 hours post-T0, data was available from n=9 uncomplicated sepsis and n=13 severe sepsis/septic shock, at 3-6 hours post-T0, data was available from n=8 uncomplicated sepsis and n=15 severe sepsis/septic shock and at 12-30 hours post-T0, data was available from n=5 uncomplicated sepsis and n=7 severe sepsis/septic shock.

# results are presented as Calculated Normalised Relative Quantity (CNRQ)
